# SR9009 Regulates Acute Lung Injury in Mice Induced by Sepsis

**DOI:** 10.1155/2022/5802938

**Published:** 2022-07-01

**Authors:** Ming Hu, Li Zhang, Jie Cao, Yu Jiang, Gang Liu

**Affiliations:** Department of Critical Care Medicine, Chongqing Medical University, Chongqing 400016, China

## Abstract

Rev-Erb*α* is a nuclear heme receptor, transcriptional repressor, and critical component of the molecular clock that drives daily rhythms of metabolism. However, the roles of Rev-Erb*α* in acute lung injury (ALI) remain unclarified. Hence, the effect of Rev-Erb*α* on lung injury of sepsis mice is investigated here. The mice sepsis model is established using lipopolysaccharide (LPS) injection, and the expression levels of proinflammatory cytokines, such as tumor necrosis factor alpha (TNF-*α*), interleukin-6 (IL-6), and interleukin-10 (IL-10) in both RAW246.7 cells and lung tissues, are tested. The inflammatory response is obviously enhanced in LPS-constructed sepsis mice and alleviated by SR9009 agonist treatment. Cell-based experiments reveal that pharmacological activation of Rev-Erb*α* via SR9009 attenuates the LPS-induced inflammatory response by suppressing TLR4-regulated NF-*κ*B activation. Sepsis induces the increase in W/D ratio; promotes the levels of malondialdehyde (MDA), lactic acid (LA), and superoxide dismutase (SOD); and inhibits the levels of glutathione (GSH), whereas SR9009 treatment could effectively yield beneficial effects on metabolism. In addition, SR9009 treatment ameliorates acidosis and hypoxemia by efficiently decreasing arterial PaCO_2_ and increasing arterial PaO_2_, SO_2_, HCO_3_^–^, lactic acid concentration, and blood PH. These findings confirm that SR9009 treatment can alleviate the sepsis-induced lung injury and targeting Rev-Erb*α* may represent a promising approach for the prevention and management of ALI.

## 1. Introduction

Sepsis, an intractable clinical syndrome, is often accompanied by multiple organ dysfunction, and the most affected organ is the lung; thus, acute lung injury (ALI) is one of the common complications of sepsis [1, 2]. What plays a core and fundamental role in sepsis-induced lung injury is the imbalance of immune response and uncontrolled inflammatory response [3]. The nuclear heme receptor Rev-Erb*α* is one of the unique and evolutionarily conserved superfamilies of ligand-regulated transcription factors that drives daily rhythms of behavior, metabolism, and inflammatory-immune responses as well as represents attractive therapeutic targets for the treatment of a variety of diseases [4–6]. Accumulating evidence reveals that Rev-Erb*α* negatively regulates the expression of various proinflammatory molecules such as the cytokine interleukin-6 (IL-6) and the chemokine CCL2, following the binding of the synthetic Rev-erb-specific agonists SR9009 and GSK4112 in macrophages [7]. Considering the crucial role played by Rev-Erb*α*, its pharmacological modulations may be a strategy for treating inflammation. The role of Rev-Erb*α* activation in anti-inflammatory regulation has been confirmed in inflammatory diseases such as colitis [8] and osteoarthritis [9]; however, whether Rev-Erb*α* affects the inflammatory response of acute lung injury caused by sepsis remains unresolved.

SR9009, a Rev-Erb*α* agonist, has been confirmed that can significantly alleviate the lipopolysaccharide (LPS) induced production of proinflammatory cytokines in the human endometrial stroma cells (hESCs) [10] and reduce the mortality of mice with fatal sepsis and effectively improve the clinical symptoms of acute pancreatitis, viral myocarditis, and other diseases [11, 12]. However, whether Rev-Erb*α* activation by SR9009 can alleviate the proinflammatory cytokines in septic ALI mice and its possible mechanism are not yet clear.

In this study, we investigated whether Rev-Erb*α* activated by SR9009 could alleviate lung injury in sepsis mice. Furthermore, this study was aimed to figure out the role of Rev-Erb*α* in ALI-related inflammation caused by sepsis in vitro and in vivo, identifying the potential link between Rev-Erb*α* and lung injury inflammation.

## 2. Materials and Methods

### 2.1. Cell Culture and Treatment

RAW246.7 mouse macrophage was obtained from the Cell Bank of American Type Culture Collection (ATCC). The cells were cultured in RPMI 1640 medium containing 10% FBS, 100 U/Ml penicillin, and 100 *μ*g/mL streptomycin. All cells were incubated at 37°C in a humidified atmosphere with 5% CO_2_ for 24 h in preparation for other treatments. For the construction of the cell model of sepsis-induced injury, RAW246.7 cells were treated with 3 *μ*g/ml LPS (L2630; Sigma) for 24 h (LPS group). For SR9009 studies, RAW246.7 cells were incubated with a complete medium containing DMSO or SR9009 (final concentration, 10 *μ*M; HY-16989; MedChemExpress, New Jersey, USA) for 2 h in the control group and SR9009 group, respectively. To clarify the role of SR9009, a Rev-Erb*α* antagonist SR8278 was adopted for comparative study (SR8278 group; HY-16989; MedChemExpress, New Jersey, USA). SR9009 group and SR8278 group need to be treated with LPS again (SR9009+LPS group and SR8278+LPS group). All the RAW246.7 cells of the different group were cultured to collect supernatant for testing the difference in cytokine protein levels.

### 2.2. Lipopolysaccharide (LPS) Induced Mice Sepsis Model

Twenty-four male BALB/C mice (6–8 weeks old, 18–20g, purchased from the Experimental Animal Center of Chongqing Medical University) were divided randomly into four groups (*n* = 6): control group, LPS group, SR9009 group, and LPS + SR9009 group. Mice in the control group were intraperitoneally injected with normal saline (20 mg/kg); those in the LPS group were injected with an equal dose of LPS; and those in the SR9009 group were injected with 50 mg/kg of SR9009. Prior to LPS induction, mice in LPS + SR9009 group were treated with SR9009 (50 mg/kg) by intraperitoneal injection. After 30 minutes, 20 mg/kg of LPS were further injected. After 16 h injection, all mice were used for follow-up experiments.

### 2.3. Hematoxylin-Eosin (H&E) Staining

The right middle lung lobes were fixed in 4% paraformaldehyde and disposed with dehydration, clearing, wax immersion, and paraffin sectioning routinely. Four micrometer tissue sections were stained with hematoxylin and eosin and placed under a light microscope to observe pathological changes in lung tissue.

### 2.4. Detection of Wet-to-Dry (W/D) Ratio of Lung Tissue

The right upper lung lobe was taken, and the blood on the surface of the lung tissue was sucked dry with filter paper. Then, the wet weight of the right upper lung lobe was immediately weighed. Lung tissue was put into the 80°C oven to be dried for 48 h. Then lung tissue was taken out, and the dry weight was immediately weighed. The wet/dry weight ratio (W/D) was calculated.

### 2.5. ELISA Assay

Cell: RAW246.7 cells were treated with LPS, SR9009, SR8278, SR9009 + LPS, and SR8278 + LPS, respectively, and then, the supernatant was absorbed, and the levels of IL-6, TNF-*α*, and IL-10 were determined according to the instructions of mouse IL-6 ELISA kit (EMC004.48, Xinbosheng), mouse TNF-*α* ELISA kit (EMC102a, Xinbosheng), and mouse IL-10 ELISA kit (EMC005.48, Xinbosheng). The standard hole, sample hole, and blank hole were set during the ELISA assay. The absorbance value was detected at the wavelength of 450 nm by a Synergy^TM^ 2 Multi-Function Microplate Reader (Biotek, Vermont, USA). The levels of malondialdehyde (MDA), superoxide dismutase (SOD), lactic acid (LD), and glutathione (GSH) in the supernatant of RAW246.7 were detected by the mouse MDA ELISA kit (KB4123, LuoMin Biotech Co. Ltd., Shanghai, China), SOD (A001-3-2, Jiancheng Tech Co. Ltd., Nanjing, China), LD (A019-2-1, Jiancheng Tech Co. Ltd., Nanjing, China), and GSH (GA-E3985RT, GenAsia Biotech Co. Ltd.).

Tissue: Appropriate amount of left lung tissue was cut and weighed. The ratio of adding 9 ml PBS to 1 g lung tissue and the ratio of adding 200 *μ*l RIPA lysates to 20 mg of lung tissue was allocated. The 10% lung homogenate was prepared on ice with a homogenizer and centrifuged at 4,400 rpm for 20 min. The supernatant was absorbed and stored at −80°C. The levels of IL-6, TNF-*α*, IL-10, MDA, superoxide SOD, LD, and GSH in the supernatant of lung homogenate were detected by mouse IL-6 ELISA kit (EMC004.48, Xinbosheng), mouse TNF-*α* ELISA kit (EMC102a, Xinbosheng), mouse IL-10 ELISA kit (EMC005.48, Xinbosheng), mouse MDA ELISA kit (KB4123, LuoMin Biotech Co. Ltd., Shanghai, China), SOD (A001-3-2, Jiancheng Tech Co. Ltd., Nanjing, China), LD (A019-2-1, Jiancheng Tech Co. Ltd., Nanjing, China), and GSH (GA-E3985RT, GenAsia Biotech Co. Ltd.).

### 2.6. Real-Time Quantitative PCR

Total RNA was extracted using the BIOZOL agent according to the manufacturer's protocol. The complementary DNA was synthesized using a reverse transcription kit (TOYOBO, Shanghai) and then amplified using specific primers as listed in Table 1, and SYRB Green PCR Master Mix was used to obtain the threshold cycle (CT) values of the genes. Real-time quantitative PCR (qRT-PCR) was conducted to denature DNA strands followed by 95°C (30 s), 95°C (5 s), 55°C (10 s), and 72°C (15 s) for 40 cycles.

### 2.7. Arterial Blood Gas Analysis of ALI Mice

At 16 h after saline, LPS, SR9009, and LPS + SR9009 injection, mice were anesthetized, and blood was drained from the abdominal aorta into a heparinized syringe and immediately used for analysis. Arterial blood analysis was performed with an IL1640 blood gas analyzer (Instrumentation Laboratory System, Lexington, MA, USA). For each mouse, five values are used in ABG analysis: pH, oxygen partial pressure (PaO_2_), carbon dioxide partial pressure (PaCO_2_), oxygen saturation (SO_2_), lactic acid (LD) concentration, and arterial bicarbonate (HCO_3_^–^) concentration.

### 2.8. Flow Cytometry Analysis for RAW246.7 Phenotype

The pretreated RAW246.7 cells were harvested and washed, and cells suspension was adjusted to a concentration of 1–5 × 10^6^ cells/ml in ice-cold PBS, 10% FCS, and 1% sodium azide. And then RAW246.7 is centrifuged sufficiently so the supernatant fluid can be removed. Add 1–2 *μ*l of the primary labeled antibody (APC anti-mouse CD206 (MMR) antibody (141708) and APC anti-mouse CD86 antibody (105012), BioLegend, USA) and incubate for at least 30 min at room temperature or 4°C. Wash the cells 3 times by centrifugation at 400 g for 5 minutes and resuspend them in 500 µl of ice-cold PBS, 10% FCS, and 1% sodium azide. Finally, analyze the cells on the flow cytometer.

### 2.9. Statistical Analysis

SPSS 20.0 statistical software was used for analysis and processing, and the measured data was represented by mean ± SD. One-way analysis of variance was used for comparison between groups, and *p* < 0.05 was regarded as a significant difference.

## 3. Results

### 3.1. SR9009 Regulated Inflammation by Mediating the Macrophages Polarization from M1 to M2

**Figure 1:**
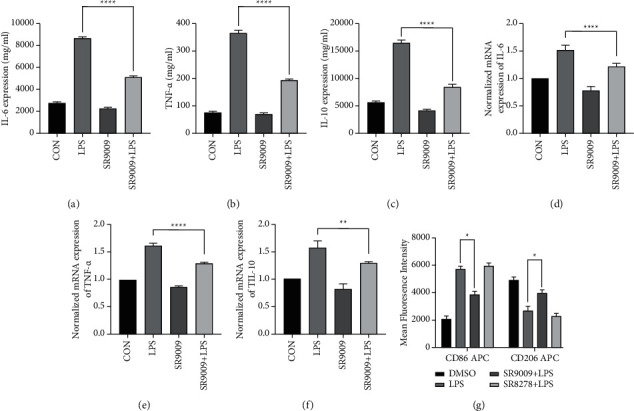
ELISA detection of proinflammatory cytokine production: (a) IL-6, (b) TNF-*α*, and (c) anti-inflammatory cytokine production IL-10 in RAW246.7 cells after treatment with DMSO (control group), LPS, SR9009, and SR9009 + LPS, respectively. Normalized mRNA expressions of (d) IL-6, (e) TNF-*α*, and (f) IL-10 in RAW246.7 cells after treatment with DMSO (control group), LPS, SR9009, and SR9009 + LPS, respectively. (g) Mean fluorescence intensity of CD86 and CD206 in RAW246.7 before and after Rev-erb*α* regulation. Significant differences:  ^*∗*^*p* < 0.05,  ^*∗*^ ^*∗*^*p* < 0.01,  ^*∗*^ ^*∗*^ ^*∗*^*p* < 0.001, and ^*∗∗∗∗*^*p* < 0.0001.

The treatment strategy of SR9009 prior to LPS treatment was adopted in our all experiments. LPS is a critical virulence component of gram-negative bacteria and was used to induce inflammatory responses [13]. IL-6 and TNF-*α* are known as major proinflammatory cytokines. We detected the relative expressions of IL-6, TNF-*α*, and IL-10 in RAW246.7 cells with treatment of LPS at a concentration of 500 ng/ml. As shown in Figure 1, LPS treatment modulated inflammatory responses obviously and presented an upregulation of proinflammatory cytokines (IL-6, Figure 1(a); TNF-*α*, Figure 1(b)). While SR9009 administration could significantly decrease the expressions of proinflammatory cytokines. We also noticed other cytokine genes that were differentially regulated by SR9009 treatment, including IL-10. As shown in Figure 1(c), LPS induced the high expression of IL-10, while SR9009 prior to LPS treatment efficiently repressed the expression of IL-10, which is consistent with Amir's work [14]. The mRNA expressions results of the IL-6, TNF-*α*, and IL-10 were consistent with the ELISA experiments.

In addition, we monitored the in situ change in the RAW246.7 phenotype using flow cytometry, as shown in Figure 1(g). As expected, the M1 protein marker CD86 was inhibited in the group of SR9009 + LPS, whereas the M2 protein marker CD206 was activated, indicating that M1 began to polarize towards M2. These results indicated that SR9009 could regulate inflammation, and the effect was conducted by mediating the macrophages polarization from M1 to M2.

### 3.2. SR9009 Mediated the Macrophages Polarization by Regulating the Expression of Rev-Erb*α*

**Figure 2:**
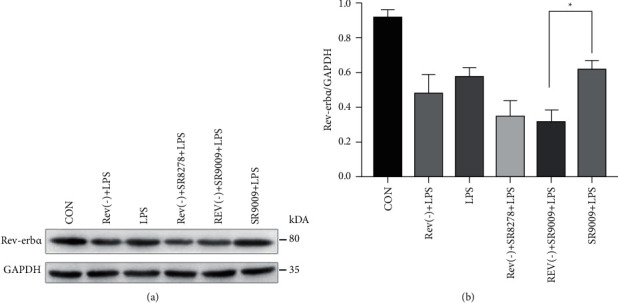
(a) Western blot analysis of Rev-Erb*α* in normal and Rev-Erb*α* knock-down RAW246.7 cells treated with DMSO, LPS, SR9009, SR9009 + LPS, and SR8278 + LPS and (b) Rev-Erb*α*/GAPDH ratio in RAW246.7 cells after LPS induction and SR9009 and SR8278 treatment. Significant differences: ^*∗*^*p* < 0.05.

It remains unclear whether the circadian rhythm is disrupted in RAW246.7 cells after LPS treatment. Thus, Rev-Erb*α* in RAW246.7 cells was knocked down by using Rev-Erb*α* sgRNA1 in advance and further detected after treatment with LPS, SR9009, and SR8278. The relevant results are presented in Figure 2. As shown in Figures 2(a) and 2(b), we found that LPS-treated RAW246.7 (without knock-down) showed a decreased Rev-Erb*α* protein level in comparison with the control group and immediately increased after further treatment with SR9009. However, the Rev-Erb*α* protein level could not be elevated by LPS in Rev-Erb*α* knock-down group (Rev(−)). Similar results also presented in groups of (Rev(−)) + SR8278 + LPS and (Rev(−)) + SR9009 + LPS. The above results indicated that SR9009 as Rev-erb-specific agonists could significantly promote Rev-Erb*α* expression in LPS-stimulated normal RAW246.7 cells and hardly work on Rev-Erb*α* knock-down” cells.

### 3.3. SR9009 Suppressed Inflammation by Regulating the Expression of Rev-Erb*α*

**Figure 3:**
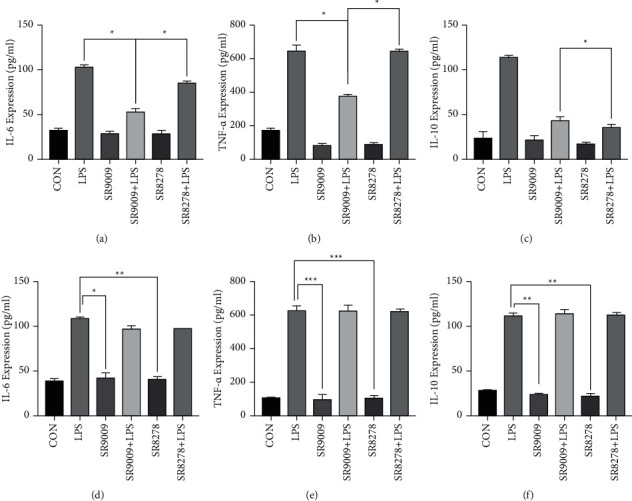
The analysis of the relative expression of Rev-Erb*α* activation by SR9009 and SR8278 in vitro. ELISA detection of proinflammatory cytokine production: (a) IL-6, (b) TNF-*α*, and (c) anti-inflammatory cytokine production IL-10 in RAW246.7 cells after treatment with DMSO (control group), LPS, SR9009, SR9009 + LPS, SR8278, and SR8278 + LPS; knock-down of Rev-Erb*α* in RAW246.7 cells aggravated the inflammatory response: (d) IL-6, (e) TNF-*α*, and (f) IL-10 in RAW246.7 cells. Significant differences:  ^*∗*^*p* < 0.05,  ^*∗*^ ^*∗*^*p* < 0.01, and  ^*∗*^ ^*∗*^ ^*∗*^*p* < 0.001.

In order to explore the effects of Rev-Erb*α* on the inflammatory response of RAW246.7 cells, two different treatment strategies of SR9009 (Rev-Erb*α* agonist) and SR8278 (Rev-Erb*α* antagonist) were adopted. As expected, SR9009 prior to LPS treatment resulted in significantly reduced IL-6 and TNF-*α* expression levels, whereas SR8278 prior to LPS treatment showed no effects, especially in TNF-*α* expression (Figures 3(a)–3(c)).

To further explore the role that Rev-Erb*α* plays in inflammation, we detected the expressions of inflammation cytokines in Rev-Erb*α* knock-down RAW246.7 cells. As shown in Figures 3(d)–3(f), when Rev-Erb*α* in RAW246.7 cells was knocked down, the relative expression levels of IL-6, TNF-*α*, and IL-10 were significantly increased. It can be seen that the activation of Rev-Erb*α* function suppressed LPS-induced proinflammatory activity.

### 3.4. SR9009 Improved Acute Lung Injury by Inhibiting the TLR4-NF-*κ*B Pathway

**Figure 4:**
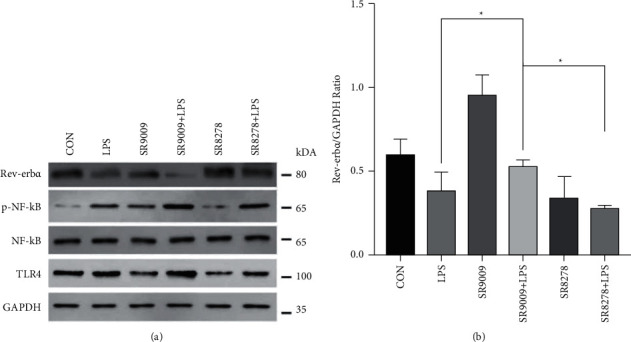
(a) Western blot analysis of Rev-Erb*α*, NF-*κ*B, and TLR4 expression in RAW246.7 treated with DMSO, LPS, SR9009, SR9009 + LPS, SR8278, and SR8278 + LPS and (b) Rev-Erb*α*/GAPDH ratio in RAW246.7 after LPS induction and SR9009 and SR8278 treatment. Significant difference: ^*∗*^*p* < 0.05.

A series of experiments above were performed to explore whether SR9009 can regulate Rev-Erb*α*. However, the potential regular mechanism of SR9009 on Rev-Erb*α* is not clear. TLR4 can recognize exogenous ligands such as LPS and plays an important role in inflammatory responses [15] Thus, we explored the protein expression of TLR4 through western blot analysis and found that LPS increased TLR4 protein expression, whereas SR9009 downregulated TLR4 protein expression induced by LPS (Figures 4(a) and 4(b)). To further explore the mechanism responsible for TLR4 signaling, we determined the activation of the NF-*κ*B pathway. The expression of NF-*κ*B showed no significant difference after treatment with LPS or SR9009. However, the expression of p-NF-*κ*B showed a similar trend to that of TLR4 expression. In conclusion, SR9009 was supposed to alleviate inflammation induced by LPS by inhibiting the TLR4-NF-*κ*B pathway. Herein, we only used SR9009 to prove its therapeutic effect on ALI in subsequent animal experiments.

### 3.5. SR9009 Ameliorated Oxidative Stress in RAW246.7 Cells

**Figure 5:**
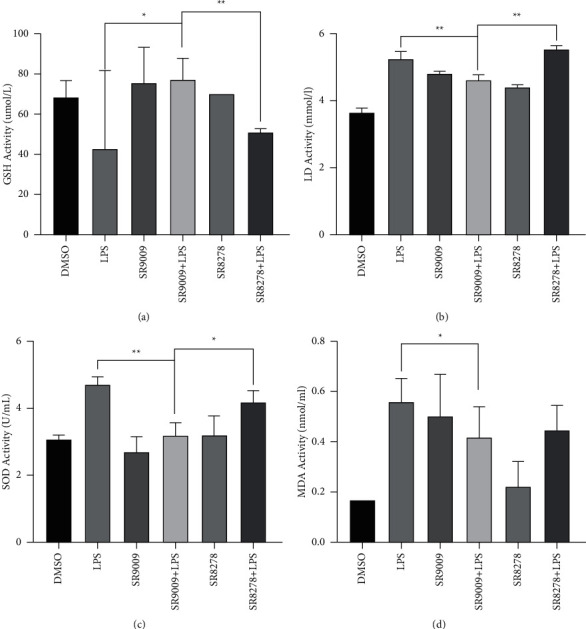
LPS-induced level of oxidative stress in RAW246.7 cells. The expression levels of GSH (a), LD (b), SOD (c), and MDA (d) were detected by ELISA assay.  ^*∗*^*p* < 0.05 and  ^*∗*^ ^*∗*^*p* < 0.01.

As shown in Figure 5, the expression levels of SOD, MDA, and LD were decreased, while  ^*∗∗*^*p* < 0.01 GSH expression level was increased in SR9009 + LPS-treated RAW 246.7 cells in contrast with the DMSO group, which is consistent with our expected results. The in vitro results will provide a theoretical basis for the subsequent in vivo experiments.

### 3.6. SR9009 Improved Acute Lung Injury Induced by Sepsis

**Figure 6:**
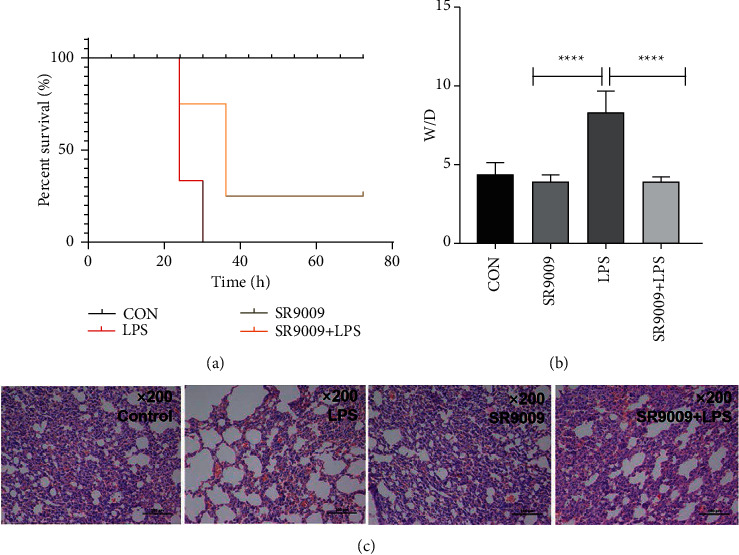
Effect of sepsis on lung tissue after SR9009 treatment: (a) the survival rate of rats after LPS, SR9009, SR9009 + LPS, SR8278, and SR8278 + LPS treatment; (b) the ratio of W/D was changed after LPS, SR9009, and SR9009 + LPS treatment; and (c) the lung histopathology of mice after LPS, SR9009, and SR9009 + LPS treatment was observed by H&E staining.  ^*∗*^ ^*∗*^ ^*∗*^ ^*∗*^*p* < 0.0001.

Mice in the control group and SR9009 group were all survived (Figure 6(a)). LPS-treated mice died in 30 hours, while the SR9009 + LPS-treated group had a survival rate of 70% in the same period. As shown in Figure 6(b), the W/D ratio of mice in the LPS group was significantly increased, and the W/D ratio was decreased when LPS-induced sepsis mice were pretreated with SR9009. The lung histopathology of mice was observed by H&E staining, as shown in Figure 6(c). In the control group and SR9009 group, the structure of lung tissues in mice was intact, and no inflammatory cells were observed. In the LPS group, diffuse hemorrhage and edema could be seen in the lung tissue of mice, accompanied by a large amount of inflammatory cells. In the SR9009 + LPS group, the pathological lesions such as hemorrhage and edema in the lung tissue of mice were relieved, and the infiltration of inflammatory cells was significantly reduced.

**Figure 7:**
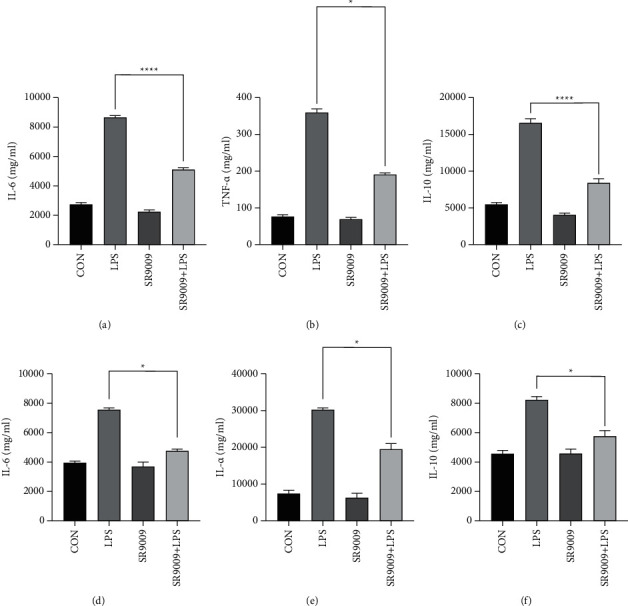
SR9009 alleviates sepsis-induced lung inflammation in mice: (a)–(c) the expression levels of IL-6, TNF-*α*, and IL-10 in lung tissues were detected by ELISA assay and (d)–(f) the expression level of IL-6, TNF-*α*, and IL-10 in serum were detected by ELISA assay. ^*∗*^*p* < 0.05.

ELISA assay results in Figures 7(a) and 7(b) and Figures 7(d) and 7(e), respectively, showed that the expression levels of IL-6 and TNF-*α* in lung tissues and in serum were significantly upregulated in LPS-induced sepsis mice, while in the SR9009 + LPS group, both IL-6 and TNF-*α* expression levels were decreased sharply, illustrating that SR9009 pretreatment could inhibit inflammation. As we expected, the expression levels of the anti-inflammatory cytokines IL-10 both in lung tissues and serum were minor increased after SR9009 pretreating in contrast to the SR9009 alone group (Figures 7(c) and 7(f)).

**Figure 8:**
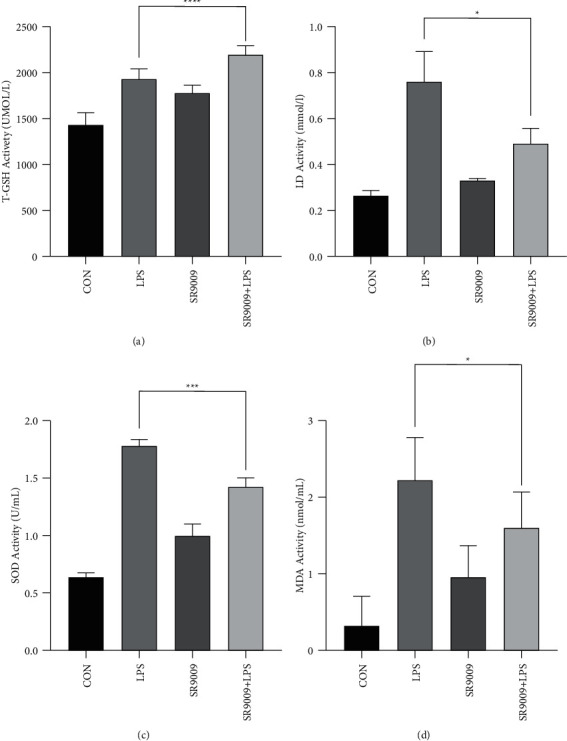
SR9009 alleviates sepsis-induced lung inflammation in mice. The expression levels of T-GSH (a), LD (b), SOD (c), and MDA (d) in lung tissues were detected by ELISA assay.  ^*∗*^*p* < 0.05,  ^*∗*^ ^*∗*^*p* < 0.01, and  ^*∗*^ ^*∗*^ ^*∗*^*p* < 0.001.

In Figure 8, the expression levels of SOD, MDA, and LD were decreased, while the T-GSH expression level was increased in sepsis-induced mice, which has the trend as same as in vitro results. To some degree, SR9009 pretreatment could reverse the expression levels of SOD, MDA, and LD in sepsis-induced mice.

Arterial blood gas [16] analysis assesses the adequacy of ventilation, oxygenation, and the acid-base status of the body by measuring the levels of pH, oxygen, carbon dioxide, and bicarbonate in arterial blood. The values of carbon dioxide and oxygen—expressed as the partial pressure of carbon dioxide (PaCO_2_) and the partial pressure of oxygen (PaO_2_)—are important in the diagnosis and treatment of patients with pulmonary and other critical conditions. To establish a possible correlation between acute lung injury and SR9009 treatment on pulmonary function, we performed a blood gas analysis and assessed any alterations in gas exchange parameters.

**Figure 9:**
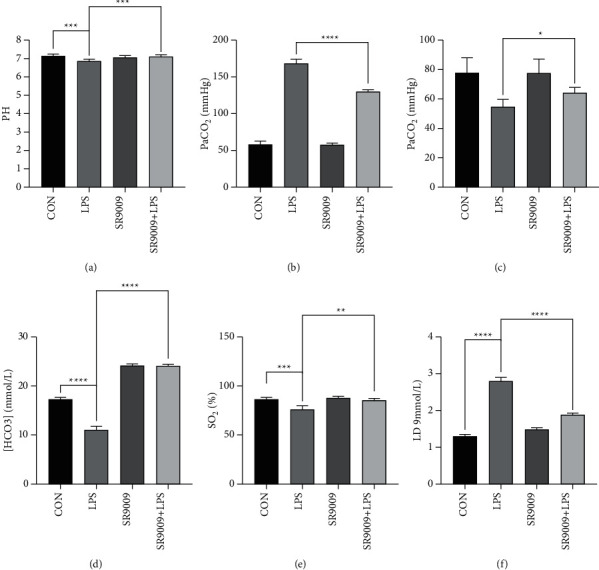
The effect of treatment on the arterial blood gas levels of different groups of mice (*n* = 10): (a)–(f) are pH, PaCO_2_, PaO_2_, HCO_3_^–^concentration, SO_2_, and LD concentration, respectively.  ^*∗*^*p* < 0.05,  ^*∗*^ ^*∗*^*p* < 0.01,  ^*∗*^ ^*∗*^ ^*∗*^*p* < 0.001, and  ^*∗*^ ^*∗*^ ^*∗*^ ^*∗*^*p* < 0.0001.

As shown in Figure 9, among the parameters monitored, arterial pH did not show significant variations in mice from the different experimental groups except the LPS group. Administration of LPS significantly increased arterial PaCO_2_, LD concentration and decreased PaO_2_, SO_2_, HCO_3_^–^ concentration. Pretreatment with SR9009 injection attenuated the changes in PaCO_2_, PaO_2_, SO_2_, LD concentration, and HCO_3_^–^ concentration.

## 4. Discussion

Recent studies have reported that several conditions, including cancer, inflammation, and metabolic dysregulation, are related to the disruption of circadian rhythm, and as such, pharmacological modulation of circadian genes is a potential target for treating these diseases [17]. Rev-Erb*α*, a heme-binging repressor, is involved in regulating circadian rhythm, metabolism, and inflammation. In mouse colitis studies, it has been proved that the pharmacological activation of Rev-Erb*α* suppresses the inflammation induced by dextran sulfate sodium [18]. However, the relationship between Rev-Erb*α* and inflammation of ALI caused by sepsis remains unclear. This study used a murine endotoxemic model induced by administration of LPS to mimic sepsis-associated ALI in humans, enabling insights into the effect of Rev-Erb*α* on ALI.

In this study, SR9009 was found to downregulate the LPS-induced production of proinflammation cytokine IL-6 and TNF-*α* in vitro, whereas Rev-Erb*α* knockdown upregulated IL-6 and TNF-*α* expressions, which is consistent with previous researches on the inhibitory effect of Rev-Erb*α* on the transcription of IL-6 [19]. As expected, the pharmacological activation of Rev-Erb*α* via SR9009 suppressed the inflammatory response induced by LPS in RAW246.7. Moreover, we further verified the mechanism behind the effect of Rev-Erb*α* after SR9009 activation on the inflammatory response. Wang et al. proved Rev-Erb*α* activation in wild-type mice by SR9009 attenuates dextran sulfate sodium (DSS) induced colitis, whereas the protective effects are lost in Nlrp3^–/–^ and Rev-Erb*α*^–/–^ mice [8]. They confirmed that Rev-Erb*α* regulates experimental colitis through its repressive action on the NF-*κ*B/Nlrp3 axis. In our study, we observed increased TLR4 expression and NF-*κ*B phosphorylation in RAW246.7 treated with LPS. Compared with the levels in the LPS group, TLR4 expression and NF-*κ*B phosphorylation were decreased in the SR9009+LPS group. We speculated that SR9009 inhibited the inflammation response of RAW246.7 by downregulating the expression of proinflammatory cytokines via the TLR4-mediated NF-*κ*B pathway.

Moreover, pretreatment with SR9009 injection could significantly alleviate the inflammatory and histological changes produced by LPS, as well as suppress the increase in LPS-induced levels of oxidative stress in the lung. In addition, SR9009 injection ameliorated acidosis and hypoxemia by efficiently decreasing arterial PaCO_2_ and LD concentration and increasing arterial PaO_2_, SO_2_, HCO_3_^–^ concentration, and blood pH. These findings confirm the protective effect of SR9009 injection in ALI, suggesting Rev-Erb*α* may attenuate inflammatory cell sequestration and migration into lung tissue. However, both in vitro and in vivo results showed that the LPS-treated group upregulated the expression of IL-10, whereas the SR9009 pretreated group could repress the expression of IL-10. IL-10 is a potent anti-inflammatory molecule that regulates excessive production of inflammatory cytokines during an infection or tissue damage. Macrophages represent a major source of IL-10, which is generated in response to TLR signaling as a feedback mechanism to curtail inflammatory response. Other researchers identified a signaling pathway in murine bone-marrow-derived macrophages in which activation of TLR4 by LPS induces the expression of IL-10 through the sequential induction of type I IFNs followed by induction and signaling through IL-27 [20], which may be related to the high expression of IL-10 induced by LPS in our study. Repression of IL-10 after SR9009 pretreatment is consistent with previously published reports demonstrating that Rev-Erb*α* directly and indirectly regulates its expression [21–23].

We have already confirmed that Rev-Erb*α* has an inflammatory regulatory effect on LPS-induced RAW246.7 cells in vitro. Thus, we speculate that Rev-Erb*α* will play the same role in ALI mice. When sepsis occurs, leukocytes become activated in the circulation, and some become lodged within the pulmonary microcirculation. Progression of the condition results in a greater accumulation of leukocytes within the lung. These leukocytes then migrate into the pulmonary interstitium, increasing capillary permeability and leading to tissue edema and disturbance of gas exchange. In the ALI mice used in this study, we observed significant leukocyte infiltration and pulmonary edema in the lung tissue after LPS treatment. Proinflammatory cytokines, including IL-6 and TNF-*α*, play an important role in ALI [24]. Persistent elevation of proinflammatory cytokines in ALI or sepsis is associated with a more negative patient outcome [25]. In this study, we found that IL-6 and TNF-*α* expression levels in both serum and lung tissues were increased after LPS treatment. Moreover, the ratio of W/D in lung tissue was also increased in lung tissues of LPS-induced sepsis mice. On the contrary, Rev-Erb*α* can inhibit the elevation in the proinflammatory cytokines IL-6 and TNF-*α* expression, as well as effectively reverse the increase of the ratio of W/D in lung tissue. MDA, LA, and SOD expressions were increased in LPS-induced sepsis mice, and SR9009 pretreatment downregulated these expressions in this study. MDA can reflect the degree of lipid peroxidation in the body and indirectly reflect the degree of cell damage. Rev-Erb*α* reversed the expression of MDA, LD, and SOD while increasing the levels of T-GSH in LPS-induced sepsis mice.

Furthermore, in LPS-treated mice, arterial blood gas analysis revealed acidosis and hypoxemia, demonstrating lung respiratory dysfunction consistent with the clinical symptoms and pulmonary lesions of ALI. These findings indicated that this LPS mouse model successfully mimics ALI. A synthetic pyrrole derivative SR9009 that acts as Rev-Erb*α*-specific agonist exhibits potent in vivo activity on metabolism and myocardial ischemia-reperfusion [26, 27]. From this result, we speculated that SR9009 injection may be effective in the treatment of ALI. In this study, we found that pretreatment with SR9009 could significantly alleviate the inflammatory and histological changes produced by LPS in the lung. Furthermore, SR9009 pretreatment ameliorated acidosis and hypoxemia by efficiently decreasing arterial PaCO_2_, and increasing arterial PaO_2_, SO_2_, HCO_3_^–^ concentration, and blood PH. The above findings confirm the protective effect of Rev-Erb*α* in ALI, suggesting Rev-Erb*α* may attenuate inflammatory cell sequestration and migration into lung tissue.

## 5. Conclusion

In summary, we found that SR9009 has a pulmonary protective effect during ALI in mice, a novel finding that has not previously been reported. The present results suggest that Rev-Erb*α* after activation meliorates ALI by suppressing a dramatic reduction in inflammatory cell infiltration and proinflammatory cytokine expression in lung tissue. In addition, the protective effect of Rev-Erb*α* in ALI is found that SR9009 pretreatment ameliorated acidosis and hypoxemia through efficiently decreasing arterial PaCO_2_, LD concentration and increasing arterial PaO_2_, SO_2_, HCO_3_^–^ concentration, and blood PH. Rev-Erb*α* servers as an integrator of colon clockwork and ALI. Targeting Rev-Erb*α* may represent a promising approach for the prevention and management of ALI.

## Figures and Tables

**Table 1:** qRT-PCR primers.

Gene name	Forward primer (5′-30)	Reverse primer (5′-30)
IL-6	CATGTTCTCTGGGAAATCGTG	TCCAGTTTGGTAGCATCCATC
TNF-*α*	CAGGGACCTCTCTCTAAT	CTACAACATGGGCTACAG
IL-10	ACCAGCTGGACAACATACTGC	CAAATGCTCCTTGATTTCTGG

## Data Availability

The experiment data used to support the findings of this study are included within the article.
